# Biocompatibility Evaluation of TiO_2_, Fe_3_O_4_, and TiO_2_/Fe_3_O_4_ Nanomaterials: Insights into Potential Toxic Effects in Erythrocytes and HepG2 Cells

**DOI:** 10.3390/nano13212824

**Published:** 2023-10-25

**Authors:** Luis Paramo, Arturo Jiménez-Chávez, Iliana E. Medina-Ramirez, Harald Norbert Böhnel, Luis Escobar-Alarcón, Karen Esquivel

**Affiliations:** 1 División de Investigación y Posgrado, Facultad de Ingeniería, Universidad Autónoma de Querétaro, Cerro de las Campanas, Santiago de Querétaro 76010, Mexico; luissofonsolaps@gmail.com; 2 Departamento de Toxicología, Centro de Investigación y de Estudios Avanzados del IPN (CINVESTAV-IPN), Ciudad de Mexico 07360, Mexico; jajimenez.c@outlook.com; 3 Departmento de Química, Universidad Autónoma de Aguascalientes, Aguascalientes 20131, Mexico; iemedina@correo.uaa.mx; 4 Centro de Geociencias, Universidad Nacional Autónoma de México, Blvd. Juriquilla, 3001, Santiago de Querétaro 76230, Mexico; hboehnel@geociencias.unam.mx; 5 Departamento de Física, Instituto Nacional de Investigaciones Nucleares, Carr. México-Toluca, La Marquesa, Ocoyoacac 52750, Mexico; luis.escobar@inin.gob.mx

**Keywords:** nanotoxicity, nanomaterials, holotomography, hemolysis assay, cell viability

## Abstract

Nanomaterials such as titanium dioxide and magnetite are increasingly used in several fields, such as water remediation and agriculture. However, this has raised environmental concerns due to potential exposure to organisms like humans. Nanomaterials can cause adverse interactions depending on physicochemical characteristics, like size, morphology, and composition, when interacting with living beings. To ensure safe use and prevent the risk of exposure to nanomaterials, their biocompatibility must be assessed. In vitro cell cultures are beneficial for assessing nanomaterial–cell interactions due to their easy handling. The present study evaluated the biocompatibility of TiO_2_, Fe_3_O_4_, and TiO_2_/Fe_3_O_4_ nanomaterials thermally treated at 350 °C and 450 °C in erythrocytes and HepG2 cells. According to the hemolysis experiments, non-thermally treated NMs are toxic (>5% hemolysis), but their thermally treated counterparts do not present toxicity (<2%). This behavior indicates that the toxicity derives from some precursor (solvent or surfactant) used in the synthesis of the nanomaterials. All the thermally treated nanomaterials did not show hemolytic activity under different conditions, such as low-light exposure or the absence of blood plasma proteins. In contrast, non-thermally treated nanomaterials showed a high hemolytic behavior, which was reduced after the purification (washing and thermal treatment) of nanomaterials, indicating the presence of surfactant residue used during synthesis. An MTS cell viability assay shows that calcined nanomaterials do not reduce cell viability (>11%) during 24 h of exposure. On the other hand, a lactate dehydrogenase leakage assay resulted in a higher variability, indicating that several nanomaterials did not cause an increase in cell death as compared to the control. However, a holotomographic microscopy analysis reveals a high accumulation of nanomaterials in the cell structure at a low concentration (10 µg mL^−1^), altering cell morphology, which could lead to cell membrane damage and cell viability reduction.

## 1. Introduction

Nanomaterials (NMs) possess different positive attributes that can be exploited in various areas like medicine, electronics, and environmental remediation [1]. The constant manipulation and production of NMs raise concerns about their fate and incorporation into the environment, leading to NMs’ interactions with living beings such as plants, animals, and human beings [2,3].

Increased production of NMs such as TiO_2_ and Fe_3_O_4_ has raised concerns about their increased presence in the environments [2]. TiO_2_ possesses photocatalytic properties used in water treatment. On the other hand, Fe_3_O_4_ possesses pollutant-removal properties [4]. These materials can disrupt living beings [2]. In TiO_2_ composites, magnetite is used to add magnetic properties in order to facilitate their handling and removal [5]. Furthermore, NM properties can be modified through techniques such as heteroatomic doping. In TiO_2_ NMs, doping serves to enhance their photocatalytic properties due to the modification of their band gap energy required for photocatalytic activation [6], where this physiochemical modification can also alter NMs’ interaction with living beings [6,7].

NMs’ exposure to human beings can occur through multiple pathways, depending on how they interact with NMs. Due to NMs’ small size, dermal penetration to deep layers and their further incorporation into the circulatory system can be achieved [8]. Inhalation of NMs leads to their incorporation into the alveolar region and, subsequently, their distribution to the circulatory and lymphatic systems [8]. The intake of NMs can lead to their absorption into the circulatory system, allowing them to reach organs such as the liver or kidneys [8,9]. During their incorporation, they travel through the human body, entering different organs. NMs can interact with various types of cells, such as erythrocytes, the main cell present in the circulatory system, and are in charge of oxygen transport to other tissues and influence hemostasis and thrombosis [10,11], or hepatocytes, which make up to 60 to 80% of the liver composition. They are also related to glucose, amino acid, and lipid metabolism and the removal of exogenous compounds [12,13].

The toxicity exerted by nanomaterials is related to the physicochemical properties of nanomaterials and the environment in which they interact. NMs’ chemical composition (metals, metal oxides, polymers, or carbon-based materials) defines properties like dissolution, affinity with tissues or molecules, and redox capacity. The properties mentioned above can also be modified by coatings, doping, or residual substances and impurities [14,15]. Size and shape influence NM incorporation pathways. Spherical structures require a lower change in cell membrane binding energy, facilitating absorption, while rod-like structures depend on factors such as the entry angle and rotation, causing membrane deformations [14]. Surface properties such as surface charge also interfere in the interaction with cells, and phenomena such as agglomeration, the aggregation of NMs, the generation of protein crowns, and the absorption of lipids and polysaccharides [14,16].

In vitro tests offer various positive aspects for assessing the toxicity of nanomaterials. For instance, immortalized cell lines such as HepG2 are suitable for evaluating drug metabolism and hepatotoxicity due to their expression of liver functions such as plasma protein secretion, cholesterol, lipoprotein and triglyceride metabolism, and glycogen synthesis [17]. However, they have disadvantages, such as a limited expression of drug-metabolizing enzymes [13]. Cell viability, apoptosis, oxidative stress testing, and cell inflammation can all be assessed using in vitro techniques [18]. Microscopy techniques like optical or fluorescent microscopes can be used to examine the interactions between nanomaterials and cells [18] as well as other physical characterizations like atomic force microscopy, which shows NM distribution as well as cell mechanical properties [19,20]. Another novel technique to assess NMs’ interaction with cells is holotomographic microscopy, based on the refractive index (RI) measurement, where the RI relates the ratio of the velocity of light in a vacuum and its velocity when traveling through a specific medium [21]. Each cell component, such as organelles, possesses different RI that can be distinguished through holotomography. NMs are included due to their higher RI; with this technique, 3D images of cells can be constructed [22,23,24].

TiO_2_ NMs can cause harmful adverse effects on cells by producing reactive oxygen species (ROS) and interacting with macromolecules, intracellular organelles, and cell membranes [25]. TiO_2_ hemolysis will depend on concentration as well as NMs’ physicochemical properties. Some works have determined that TiO_2_ can cause 52.5% of hemolysis at 250 mg L^−1^ [26] and 5–36% at 50–1000 µg mL^−1^ [27]. On the other hand, some works have shown that TiO_2_ has no hemolytic effects at 40–100 µg mL^−1^ [28]. Hemolysis tests using magnetite show that under concentrations of 0.25, 0.50, and 3.0 mg mL^−1^ [29] and 12.5–100 µg mL^−1^, no hemolytic effect is caused [30].

TiO_2_ has not shown toxicity in 2D and 3D HepG2 cultures employing alamarBlue tests at 1–75 µg cm^−2^ [31]. However, at doses of 50–200 µg mL^−1^, HepG2 cells showed a substantial uptake of TiO_2_ nanoparticles (NPs) [32]. Another study using Ag-TiO_2_ nanoparticles demonstrated that cell viability seen through (3-(4,5-Dimethylthiazol-2-yl)-2,5-diphenyltetrazolium bromide) (MTT) was reduced at 25–200 µg mL^−1^. Cell viability also decreased as dopant concentration was augmented (0.5, 2.5, and 5%). On the contrary, pure TiO_2_ did not cause a cell viability decrease, as seen through MTT. However, lactate dehydrogenase (LDH) leakage was caused by doped and pure TiO_2_ [7]. In contrast, magnetite NPs did not exhibit toxicity at 25 µg mL^−1^, and in higher concentrations (400 µg mL^−1^), a 14% reduction of cell viability was observed [33]. Additionally, the MTT assay showed cytotoxic effects on HepG2 cells exposed to Fe_3_O_4_ NPs at 25, 50, and 100 µg mL^−1^ [34]. It is essential to take into account the conditions in which viability tests are driven since it is known that NMs can interfere with several types of viability assays, including colorimetric based [35], where NMs properties like absorption capacity, hydrophobicity, surface charge tend to interfere with the assays [36].

This study compares the biocompatible properties of NMs featured in water treatment, such as TiO_2_, Fe_3_O_4_, and their composite in cells related to the circulatory and hepatic system, using traditional cell viability techniques such as [3-(4,5-dimethylthiazol-2-yl)-5-(3-carboxymethoxyphenyl)-2-(4-sulfophenyl)-2H-tetrazolium] (MTS) and LDH, hemolysis assays, and holotomography. By analyzing the results, this study aims to provide insight into the biocompatibility of these NMs and how their physicochemical properties affect hemolytic activity and cell viability. This study also aims to contrast results obtained from different cell analysis techniques.

## 2. Materials and Methods

### 2.1. Synthesis of TiO_2_

TiO_2_ NMs were prepared by modifying the method reported in [37], where silica and magnetite core-shell nanoparticles were synthesized. Initially, 0.08 g of cetyltrimethylammonium bromide (CTAB) surfactant (Sigma Aldrich, St. Louis, MO, USA, ≥98%) was added to 13 mL of ethanol (Golden Bell, Zapopan, Mexico), and the solution was stirred for 20 min. Then, 0.66 mL of titanium isopropoxide (Sigma Aldrich, St. Louis, MO, USA, 97%) was added and stirred for another 20 min. Next, 27.5 mL of distilled water containing 0.5 mL of NH_4_OH at 30% *v*/*v* (Golden Bell, Zapopan, Mexico) was added to the solution slowly. The mixture was stirred for 3 h and 30 min at 60 °C. The material obtained was subjected to a reflux wash with acetone (J.T. Baker, Phillipsburg, NJ, USA) for 1 h and multiple water/ethanol (1/1) washes. Finally, the material was dried in a convection oven (Hinotek DHG-9023A, Ningbo, China) and divided for calcination (WiseTherm FP/FHP, PMI Labortechnik GmbH, Wettingen, Switzerland) at 350 °C and 450 °C for 3 h, reaching the desired temperature in 15 min.

### 2.2. Synthesis of Fe_3_O_4_

The synthesis of Fe_3_O_4_ NMs was performed based on [38]. Two solutions (0.5 M) of FeCl_3_⋅6H_2_O (J.T. Baker, Phillipsburg, NJ, USA, 99%) and FeSO_4_⋅7H_2_O (J.T. Baker, Phillipsburg, NJ, USA, 99%) were prepared and combined in a three-necked flask under an N_2_ atmosphere while subjected to an ultrasonic bath for 15 min (Branson 1510, Brookfield, CO, USA). Next, 10 mL of 30% *v*/*v* NH_4_OH (J.T. Baker, Phillipsburg, NJ, USA) was slowly added to the mixture while continuing the ultrasonic treatment for 20 min. Once the magnetite was formed, the mixture was washed with ethanol (J.T. Baker, Phillipsburg, NJ, USA, 99%) until the pH reached neutrality. The product was filtered and dried at 50 °C in a convection oven (Air expert, Froilabo, Collégien, France).

### 2.3. Synthesis of TiO_2_/Fe_3_O_4_

The composite nanomaterials were synthesized by first sonicating 3.1 mg of magnetite in 13 mL of ethanol (Golden Bell, Zapopan, Mexico) for 20 min. 0.08 g of CTAB (Sigma Aldrich, St. Louis, MO, USA, ≥98%) was added to the mixture and stirred for 20 min, then 0.66 mL of titanium isopropoxide (Sigma Aldrich, St. Louis, MO, USA, 97%) was added and stirred for another 20 min. Subsequently, 27.5 mL of distilled water with 0.5 mL of NH_4_OH (Sigma Aldrich, St. Louis, MO, USA, 28% *v*/*v*) was slowly added into the flask and stirred for 3 h. The mixture was then stirred for another 30 min under 60 °C. The sample was washed under reflux with acetone (J.T. Baker, Phillipsburg, NJ, USA) for 1 h and, subsequently, with ethanol/water washes (1/1). To prepare the Fe-TiO_2_/Fe_3_O_4_ composite, FeCl_2_·4H_2_O (Sigma Aldrich, St. Louis, MO, USA, 99%) was added to the 27.5 mL of water previously used to produce a 0.1%w doping. The material obtained was divided and calcined at 350 °C and 450 °C for 3 h, reaching the target temperature in 15 min.

### 2.4. Characterization of Nanostructured Materials

The NMs were characterized by scanning electron microscopy (SEM) using a JEOL JXA-8530F (JEOL, Tokyo, Japan) (EDS) with an acceleration voltage of 20 kV. Transmission electron microscopy (TEM) analysis was performed using JEOL JEM-1010 (JEOL, Japan) equipment. Magnetic susceptibility was determined using a Micromag 2900 Magnetometer (Lake Shore Cryotronics, Westerville, OH, USA). The crystalline structure was analyzed using a Bruker D2 Phaser X-ray powder diffractometer (Bruker, San Jose, CA, USA) with Cu Kα radiation (λ = 1.5406 nm) and complemented with Raman spectroscopy using Horiba Scientific LabRAM (Horiba, Osaka, Japan) equipment with Nd: YAG laser (λ = 532 nm). The electronic properties of the materials were determined using photoluminescence spectroscopy with the Horiba FluoroMax 4 (Horiba, Japan) equipped with a 150 W xenon lamp.

Hydrodynamic diameter and Z potential were determined with fresh suspensions of the different NMs prepared at 1 mg/mL concentration. The NMs were suspended in water, saline solution (0.9%), or DMEM medium and sonicated for 15 min in a bath sonicator. After the suspensions were ready, 1 mL of each suspension was placed in a polystyrene cell to determine the hydrodynamic size, the polydispersive index (PDI), or the zeta potential. After 24 h, only the characteristics were determined in the suspensions prepared in the DMEM medium to analyze the change in the interaction between the NMs and the medium.

### 2.5. Hemolytic Assay of Nanomaterials

Hemolysis assays were performed using heparin-stabilized human blood, as described by [39]. Moreover, 100 μL of freshly collected blood was added to test tubes with 10 mL saline solution. Different amounts of NMs previously dispersed in a 5%V arabic gum solution (Golden Bell, Zapopan, Mexico) were added according to Table S1 in the Supplementary Material, using NMs at 10, 30, 50, and 70 µg mL^−1^ concentrations. Samples were prepared in triplicates and placed in a water bath at 37 °C for 5 h. Then, the samples were centrifuged (OHAUS FC5714 Frontier 5000, Hampton, NH, USA) at 3500 rpm for 5 min. The hemoglobin amount was determined by UV-Vis spectroscopy using a Helios Omega (ThermoScientific, Waltham, MA, USA) spectrometer at 525 nm. Two different assays were carried out under light exposure and obscurity. Additionally, to eliminate interactions between proteins and NMs, a test was carried out by removing the serum package in the blood through centrifugation at 2500 rpm for 5 min and substituting the equivalent volume of serum with saline solution. Afterward, 100 μL of blood sample was added according to the methodology.

### 2.6. Cell Viability Assay

HepG2 cells obtained from the American Type Culture Collection (HB-8065) were used for viability tests. Cells were cultured in Dulbecco’s Modified Eagle Medium (DMEM) high glucose medium with 10% fetal bovine serum, 1% antibiotic, and 1% antifungal and incubated at 5% CO_2_ atmosphere and 37 °C. Cells were grown in 96-well plates with a 6 × 10^4^ cells/cm^2^ density. The NMs were dispersed in sterilized distilled water to prepare solutions at 30, 50, 70, and 90 µg mL^−1^ in DMEM medium. The medium in each well with cells was replaced with NM solutions and then incubated for 24 h. The positive control consisted of unexposed HepG2 cells.

After incubation, cell viability was measured by the MTS assay. (CellTiter 96^®^ Aqueous One Solution Cell Proliferation Assay, Promega, Madison, WI, USA) The supernatant medium was stored in another culture plate, replaced with 100 µL of MTS reagent, and then incubated for four hours at 37 °C. Once the incubation time was completed, the supernatant was transferred to a third plate, and the absorbance was measured at 450 nm using a Multiscan GO (Thermofisher, Waltham, MA, USA).

Lactate dehydrogenase was evaluated by taking 50 µL of supernatant from the plate of cells exposed to NMs and placing it on a new 96-well plate. Subsequently, 50 µL of previously prepared LDH reagent (Cytotoxicity Detection Kit (LDH), Roche, Basel, Switzerland) was added to each well. The plate was left to incubate for 30 min to be later analyzed in a plate reader at 450 nm (Multisakn GO ThermoScientific, Hampton, NH, USA). The positive control for this assay was performed with control cells lysed by scraping. Each of the MTS and LDH samples was performed in triplicate.

### 2.7. Holotomography Assay

The interaction of NMs with HePG2 cells was evaluated through holotomographic microscopy. HepG2 cells were incubated in circular glass slides (13 mm diameter) at 4 × 10^4^ cells/cm^2^ density and exposed to a concentration of 10 µg/mL of NMs. Cell samples were exposed to a fluorophore (HCS LipidTOX^TM^ Neutral Lipid Stains, Invitrogen, Waltham, MA, USA) with an affinity for lipid droplets to support their identification. Samples were analyzed using an HT-2H microscope (Tomocube Inc., Daejeon, Korea).

### 2.8. Statistical Analysis

The GraphPad Prism software (version 10.1.0) conducted a statistical analysis of cell viability results. An ANOVA test was conducted on the MTS and LDH cell viability assays where a post-treatment using a Bonferroni test was performed to find the differences between treatments based on the *p*-values: * *p* < 0.05, ** *p* < 0.01, *** *p* < 0.001 vs. untreated cells. The standard error of the mean (SED) was measured for each sample. Each concentration had a sample size of 16, including triplicates.

## 3. Results

### 3.1. Scanning Electron Micrography (SEM) and Energy Dispersive X-ray Spectroscopy (EDS)

**Figure 1:**
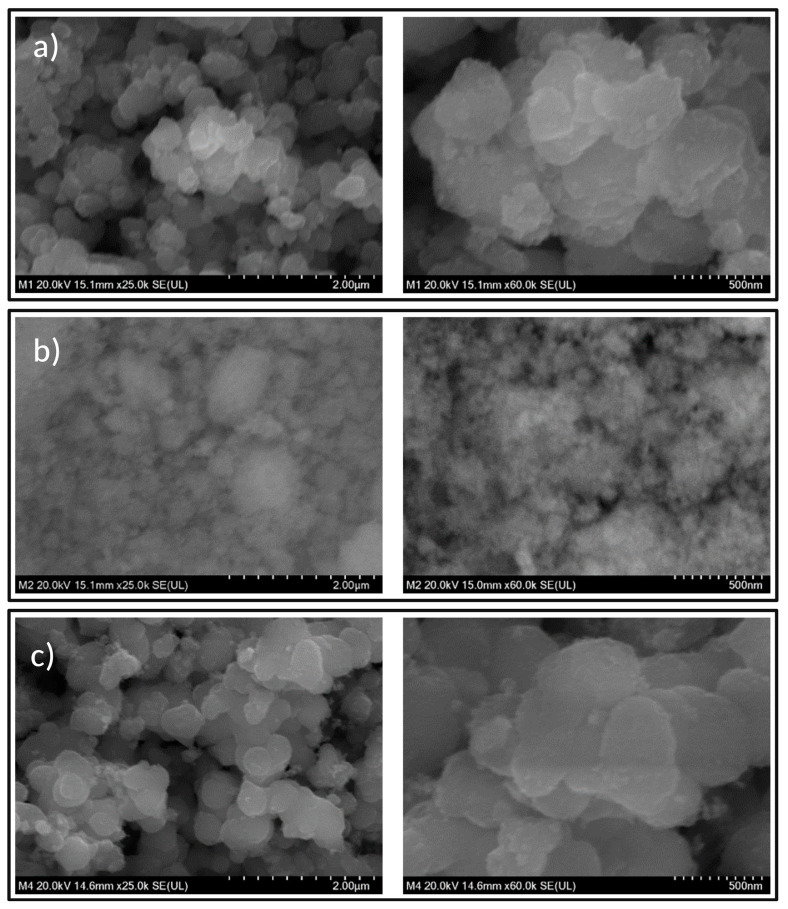
SEM micrographs of (**a**) TiO_2_, (**b**) Fe_3_O_4_, and (**c**) TiO_2_/Fe_3_O_4_.

Figure 1 shows SEM micrographs of the synthesized materials in 25,000× and 60,000× increases. The micrography of the NMs of TiO_2_ (Figure 1a) shows agglomerates of various sizes with non-geometric morphology. Similarly, Fe_3_O_4_ (Figure 1b) NMs exhibit irregular morphology with no size homogeneity, although smaller than the TiO_2_ NMs. On the other hand, the NMs composite (Figure 1c) shows morphological characteristics similar to those of TiO_2_ NMs.

**Figure 2:**
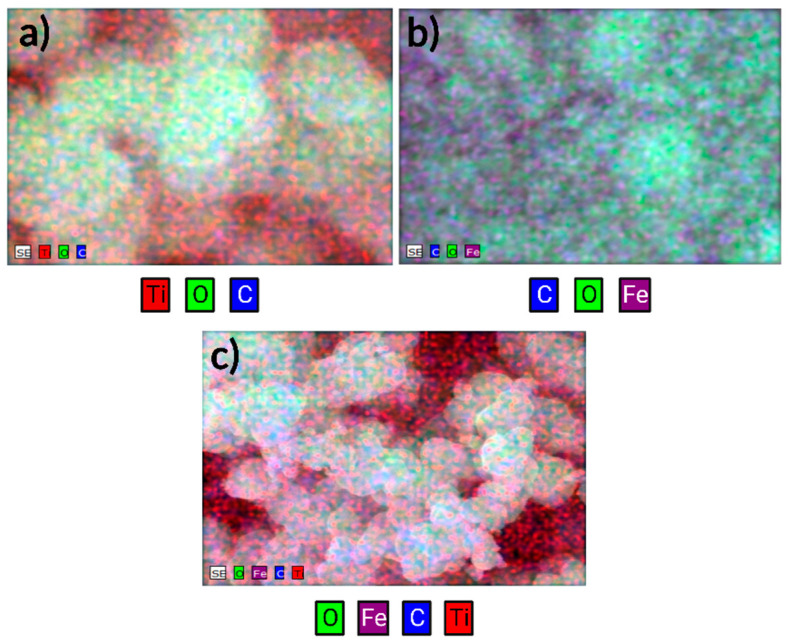
Energy dispersive X-ray elemental analysis of (**a**) TiO_2_, (**b**) Fe_3_O_4_, and (**c**) TiO_2_/Fe_3_O_4_.

The TiO_2_ sample (Figure 2a) indicates the presence of elements such as titanium, oxygen, and carbon. The presence of carbon can be attributed to surfactant residues, which were not eliminated during the washing procedure and were subjected to calcination. The mapping of the sample of Fe_3_O_4_ (Figure 2b) indicates the presence of iron, oxygen, and carbon, the latter being attributed to residues of filter paper used for drying. Finally, the composite material (Figure 2c) combines the abovementioned elements, including carbon, from calcined surfactant residue. EDX elemental spectra, as well as scanning electron microscopy mapping zones, can be seen in Supplementary Figures S1–S4.

### 3.2. Transmission Electron Microscopy (TEM)

**Figure 3:**
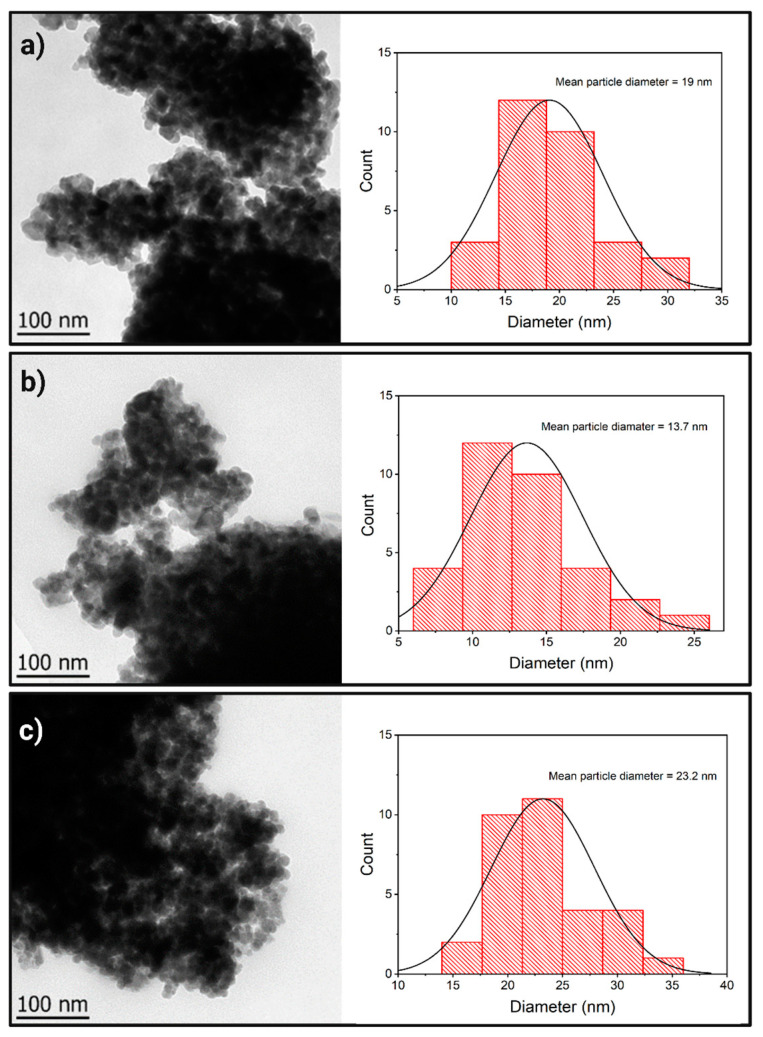
TEM micrographs and NM diameter distributions of (**a**) TiO_2_, (**b**) Fe_3_O_4_, and (**c**) TiO_2_/Fe_3_O_4_.

Electronic transmission microscopy was used to obtain greater magnification, allowing the determination of morphological characteristics and the performance of an average material size approach. The micrography corresponding to the TiO_2_ (Figure 3a) shows highly agglomerated nanoparticles with irregular morphology. The average size of these nanoparticles was determined using Feret’s diameter method [40]. TiO_2_ samples have an average diameter of 19 nm. Fe_3_O_4_ micrography (Figure 3b) shows a highly agglomerated irregular morphology with an average size of 13.7 nm.

Similarly, the composite (Figure 3c) exhibits a highly agglomerated irregular morphology with an average diameter of 23.2 nm. From TEM, it was possible to observe that the general morphology of the NMs synthesized using surfactant was not similar to the hollow spheres reported in [37]. As shown in the EDS mapping of the composite Figure 2c, the iron is present along the mapping zone, which can be interpreted as a mixture of the materials rather than a core-shell structure, which could not be confirmed through TEM. It should be clarified that the obtained distribution size is just a representative value, and techniques like DLS should be used to estimate its value correctly.

### 3.3. Crystallographic Analysis (X-ray Powder Diffraction (XRD) and Raman Spectroscopy)

**Figure 4:**
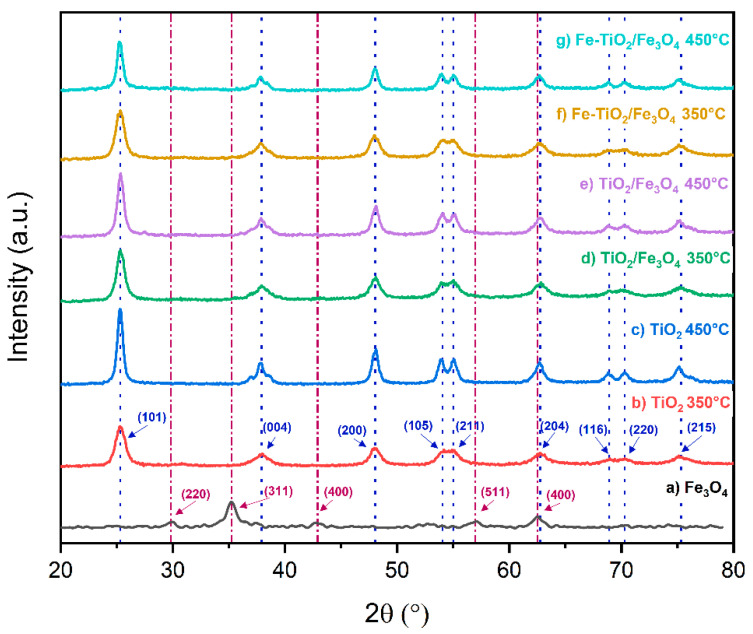
X-ray diffraction patterns of the synthesized nanomaterials.

Figure 4 shows the X-ray diffraction patterns of the synthesized materials. The diffraction pattern of TiO_2_/Fe_3_O_4_ indicates a certain degree of crystallinity, showing five characteristic Bragg reflections of the magnetite phase at angles 30.44°, 35.79°, 43.42°, 57.46°, and 63.0° (JCPDS 19-0629) [41]. The TiO_2_ material calcined at both temperatures shows nine Bragg reflections associated with the anatase phase at angles 25.1°, 37.7°, 47.8°, 53.6°, 54.8°, 62.5°, 68.7°, 70.1°, and 75.1° (JCPDS 21-1272) [42,43]. The diffraction patterns of the composites reveal the presence of the nine signals attributed to anatase without any reflections belonging to the magnetite phase due to its low percentage of TiO_2_.

Table 1 displays the crystallite size calculated by Scherrer’s and Williamson Hall’s equations [44]. The Scherrer method indicates an average size between 7 and 13 nm, while the Williamson–Hall model yields an average crystal size between 6 and 12 nm. Larger crystallite sizes were observed in treatments at 450 °C compared to 350 °C treatments.

Powder diffraction validated the synthesis of structures such as TiO_2_ in the anatase phase and Fe_3_O_4_. X-ray diffraction could not verify iron-related structures due to the low concentration used to form the composites, causing a dilution effect and therefore needing techniques such as X-ray photoelectron spectroscopy (XPS) for its detection [45].

**Figure 5:**
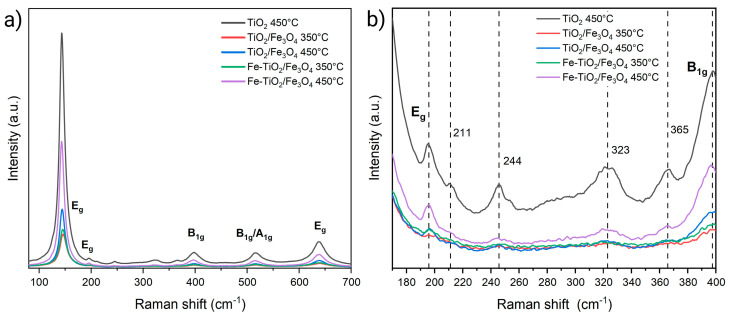
(**a**) Raman spectra of the synthesized nanomaterials and (**b**) additional vibrational modes located between 180 and 400 cm^−1^ attributed to the brookite phase.

Figure 5a presents the Raman spectrum of the TiO_2_ samples treated at 450 °C and the undoped and doped NMs treated at 350 °C and 450 °C. All materials exhibit five signals associated with anatase located at 144, 195, 638 cm^−1^ (E_g_) and 398 and 518 cm^−1^ (B_1g_/A_1g_) [46]. Besides the anatase vibrational modes, four additional signals are observed in the 180–400 cm^−1^ range, which is attributed to the brookite phase (Figure 5b) [46].

### 3.4. Magnetic Susceptibility

**Figure 6:**
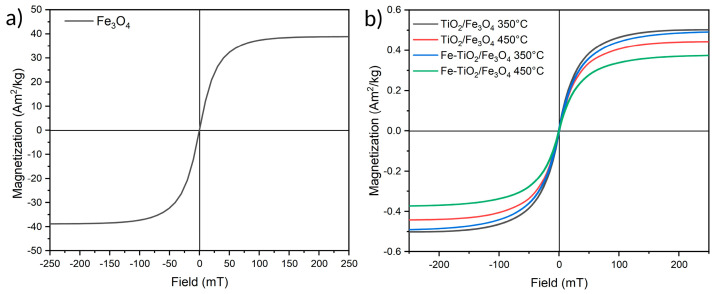
Magnetic hysteresis curves of (**a**) Fe_3_O_4_ and (**b**) TiO_2_/Fe_3_O_4_ composites.

A magnetic susceptibility test was performed on Fe_3_O_4_ and composite NMs to determine their magnetic behavior through hysteresis. Figure 6a indicates that Fe_3_O_4_ NMs have a saturation magnetization of approximately 40 Am^2^kg^−1^. The hysteresis curve shows a superparamagnetic behavior with a small ferromagnetic contribution due to particles exceeding the superparamagnetic domain boundary [38]. Figure 6b displays the hysteresis curves of the NM composites possessing a saturation magnetization range of 0.3 to 0.5 Am^2^ kg^−1^ due to a low proportion of magnetic material concerning TiO_2_. Despite the decrease in magnetization after calcination, composites treated at 350 °C and 450 °C possess a response to a magnetic field having a superparamagnetic behavior with a small ferromagnetic contribution.

### 3.5. Photoluminescence Spectroscopy (PL)

**Figure 7:**
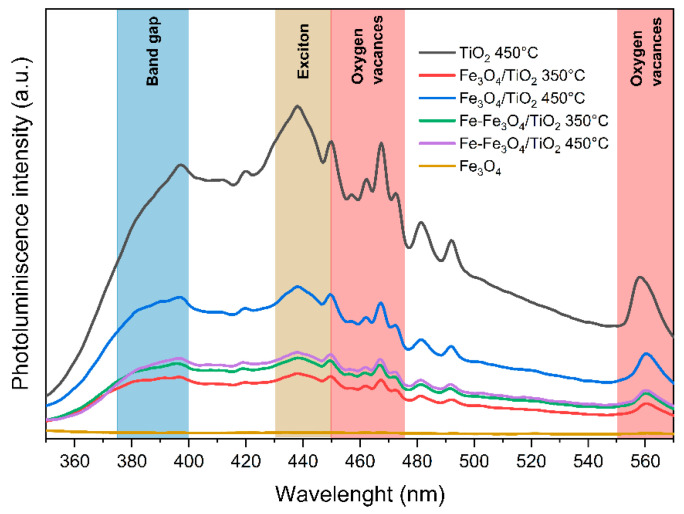
Photoluminescence spectra of the synthesized nanomaterials.

Figure 7 depicts the photoluminescence spectra of the synthesized materials, which share a similar photoluminescence spectrum but differ in signal intensity. The spectrum can be divided into four regions based on references. The first region corresponds to energetic transitions related to the bandgap (370–400 nm). The second region is associated with exciton generation processes (430–450 nm). The third region (450–475 and 550–570 nm) is linked to oxygen vacancies [47]. The intensity of these signals is correlated to exciton recombination rates, which are longer in the TiO_2_ white material synthesized at 450 °C. Composite materials exhibit a lower intensity due to the carbon and iron impurities from the magnetite, resulting in defect formation and decreasing excitons’ generation and extinction rates, thus promoting recombination processes [47].

### 3.6. NM Characterization in Suspension

Hydrodynamic diameter and Z potential were analyzed to obtain information about the NM’s stability in multiple tested media like water, saline solution (0.9%), and DMEM media. When comparing the change of hydrodynamic size and Z potential between water and saline solution (Table 2), it can be observed that the hydrodynamic size of Fe_3_O_4_ is smaller in saline solution, where a decrease in the PDI is also observed, indicating a narrower size distribution in the saline media; Z potential reveals a slight increase in saline media. TiO_2_ 350 °C in saline media possesses a larger hydrodynamic size with a reduction of PDI, indicating a narrower size distribution, while Z potential has a slight change toward a less negative charge. TiO_2_, at 450 °C in saline media, possesses a higher hydrodynamic diameter than water media, showing a slight reduction of PDI and a slight increase toward a negative charge in Z potential. Next, the composite TiO_2_/Fe_3_O_4_ 350 °C increases, and PDI reduces (narrower size distribution), while Z potential slightly decreases in negative charge. At last, the composite Fe-TiO_2_/Fe_3_O_4_ increases in hydrodynamic size when present in saline media with a slight reduction of PDI (slightly narrower size distribution) and becomes less negative in charge. Based on the Z potential of the tested materials, it is possible to infer that in water- and salt-rich media, the NM stability ranges from flocculation and coagulation (0 to ±5) to incipient instability (±10 to ±30) [48].

NMs disperse in DMEM at 0 h, and at 24 h, it is indicated that Fe_3_O_4_ NMs suffer a decrease in hydrodynamic diameter while PDI increases, displaying a slightly broader size distribution after 24 h. Z potential becomes more negative, resulting in higher stability. TiO_2_, at 350 °C, shows a slight decrease in hydrodynamic diameter and an increase in PDI, indicating a broader size distribution; Z potential also does not show a high variation between analyses. Hydrodynamic size data of TiO_2_ 450 °C show a reduction of hydrodynamic size and PDI, indicating narrower size distributions. Z potential data are only available at 24 h, having lower stability than other samples. TiO_2_/Fe_3_O_4_, at 350 °C, shows that after 24 h, NMs decrease in hydrodynamic diameter and slightly increase in PDI, while the Z potential is more negative, meaning higher stability.

At last, Fe-TiO_2_/Fe_3_O_4_ has a reduction of hydrodynamic size and an increase of PDI, resulting in a broader distribution of sizes; on the other hand, Z potential becomes more negative, indicating higher stability. Generally, it is possible to observe that Z potential after 24 h becomes more negative, except for TiO_2_ at 350 °C, which has no variation between times. The negative charge increase in Z potential could be attributed to the formation of protein corona on the surface of the NMs, as seen in gold nanoparticles exposed to poor and rich protein media [49]. It can also be observed that Z potential values in DMEM media correspond to excellent stability (greater than ±60) [48], except for TiO_2_ 350 °C.

### 3.7. Hemolysis Assay

**Figure 8:**
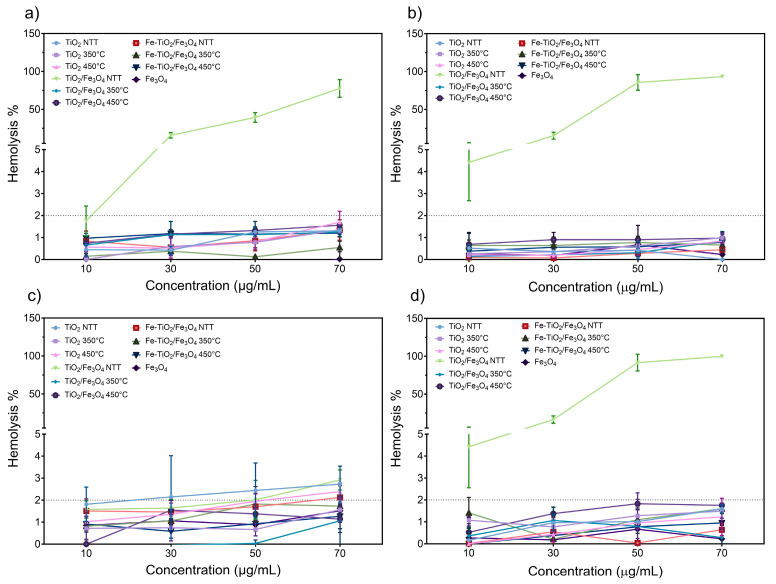
Hemolysis assay of NMs’ interaction with (**a**) blood and plasma in a dark environment, (**b**) blood and plasma under light exposure, (**c**) blood without plasma under a dark environment, and (**d**) blood without plasma under light exposure.

Figure 8 shows the results of the hemolysis tests performed on red blood cells exposed to NMs. Figure 8a corresponds to the test with blood and plasma in the dark, demonstrating that all materials remain below 2% hemolysis and are non-hemolytic. However, the non-thermally treated (NTT) NM TiO_2_/Fe_3_O_4_ composite shows increased hemolytic activity with increasing concentration. This trend is also observed in the other non-thermally treated NMs, which lose their hemolytic character after calcination. Subjecting the non-thermally treated materials to a second wash reduced the degree of hemolytic activity, as shown in Figure 8a. The hemolytic behavior is possibly attributed to surfactant residue on the material’s surface, which interacts with the red blood cell’s surface and causes hemolysis, where after calcination and CTAB removal, the hemolytic activity is lost.

The hemolysis tests conducted on NMs with plasma under light exposure are shown in Figure 8b. The results demonstrate that non-thermally treated materials have no hemolytic activity when thoroughly washed, unlike unwashed non-thermally treated materials like TiO_2_/Fe_3_O_4_. Conversely, the calcined materials did not respond consistently to light irradiation. TiO_2_ and the TiO_2_/Fe_3_O_4_ composite calcined at 450 °C showed a slight increase in their hemolytic activity due to light exposure; this could be attributed to increased electrostatic interactions or photocatalytic activity leading to ROS generation. To prove this result, photocatalytic degradation of paracetamol as a model pollutant under UV light showed that the composite NMs calcinated at 450 °C have the lowest photocatalytic activity, as seen in Figure S5 in the Supplementary Material. Nevertheless, these materials remain non-hemolytic.

The blood plasma was removed from the samples through centrifugation to eliminate the potential interference of proteins on the hemolytic activity of NMs. The results of this test are presented in Figure 8c under dark conditions, where all materials exhibit non-hemolytic activity, with non-thermally treated materials only showing hemolytic activity prior to washing. With the absence of proteins in the medium, TiO_2_/Fe_3_O_4_ calcined at 450 °C shows an increase in hemolysis compared to the other materials, indicating that removing proteins allows for greater interaction between red blood cells and NMs. However, the hemolytic activity of all materials remains below 2%.

Blood assays without plasma under light irradiation are shown in Figure 8d, indicating that non-thermally treated nanoparticles like TiO_2_ and TiO_2_/Fe_3_O_4_ have hemolytic degrees that can approach 2%, possibly caused by surfactant residue left behind after washing. In this instance, the lack of proteins and the presence of light had an overall elevating influence on the hemolytic activity of the nanomaterials. TiO_2_ at 450 °C exceeds the 2% threshold at a 70 µg mL^−1^ concentration. A complete image of hemolysis assays, including the control, can be seen in Figure S6 in the Supplementary Material.

### 3.8. Cell Viability Assay

**Figure 9:**
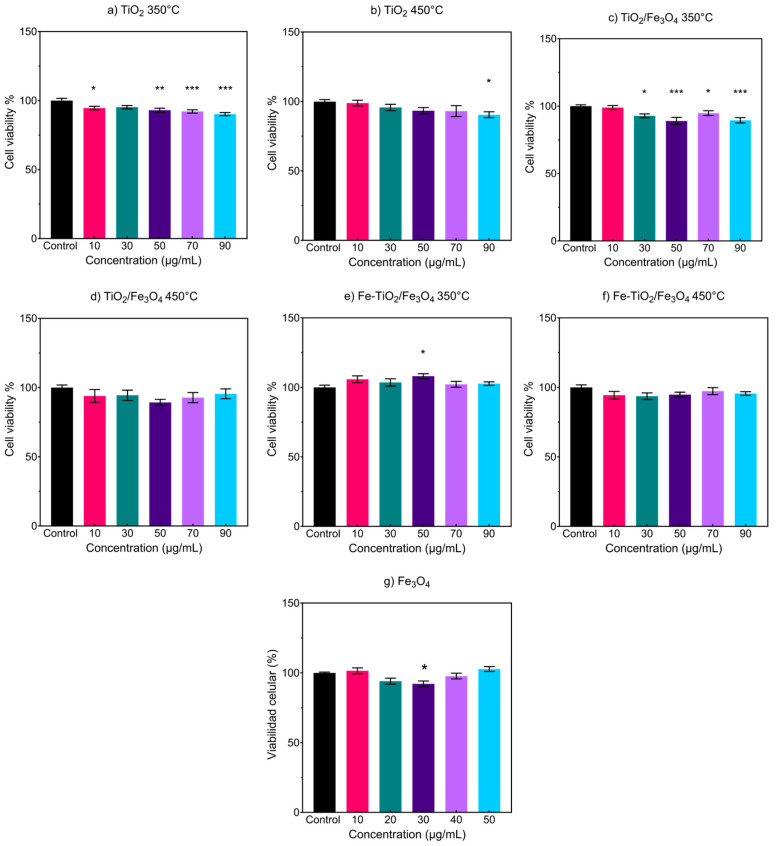
MTS cell viability assays of (**a**) TiO_2_ at 350 °C, (**b**) TiO_2_ at 450 °C, (**c**) TiO_2_/Fe_3_O_4_ at 350 °C, (**d**) TiO_2_/Fe_3_O_4_ at 450 °C, (**e**) Fe-TiO_2_/Fe_3_O_4_ at 350 °C, (**f**) Fe-TiO_2_/Fe_3_O_4_ at 450 °C, and (**g**) Fe_3_O_4_. Cell viability by MTS assay was determined after 24 h of exposure to increasing concentrations of NPs. Each bar represents the average value of 3 different experiments ± standard error of the mean (SEM); * *p* < 0.05, ** *p* < 0.01, *** *p* < 0.001 vs. untreated cells. ANOVA, Bonferroni post hoc.

MTS cell viability test graphs for each NM are shown in Figure 9; based on the results, it can be observed that TiO_2_ 350 °C (Figure 9a) causes a cell viability decrease (5.4–9.8%) with statistical significance at 10, 50, 70, and 90 µg/mL. Contrary, TiO_2_ 450 °C (Figure 9b) only shows differences in cell viability at 90 µg/mL with a reduction of 9.4%. The composite materials of TiO_2_/Fe_3_O_4_ at 350 °C (Figure 9c) caused a reduction at 30, 50, 70, and 90 µg/mL of 5.2–11%, the reduction being the highest at 50 µg/mL. The composite calcined at 450 °C (Figure 9d) shows no difference in cell viability compared to the control. The iron-doped composites (Figure 9e,f) show no effect of decreased or increased cell viability, suggesting that they do not affect cell viability at the concentrations used compared to undoped composites. Finally, the results obtained with the magnetite NMs (Figure 9g) show no trend in effect on cell viability with increasing concentration, where only the treatment at 30 µg mL^−1^ caused a reduction of 7.8%. Along the tested NMs, no apparent effect of cell viability concerning the concertation of NMs was determined. However, it is possible to observe that the main statistical differences are obtained in NMs thermally treated at 350 °C with a slight reduction of cell viability as the concentration increases. On the other hand, NMs thermally treated at 450 °C, including Fe_3_O_4_, show a lateral variation of cell viability.

**Figure 10:**
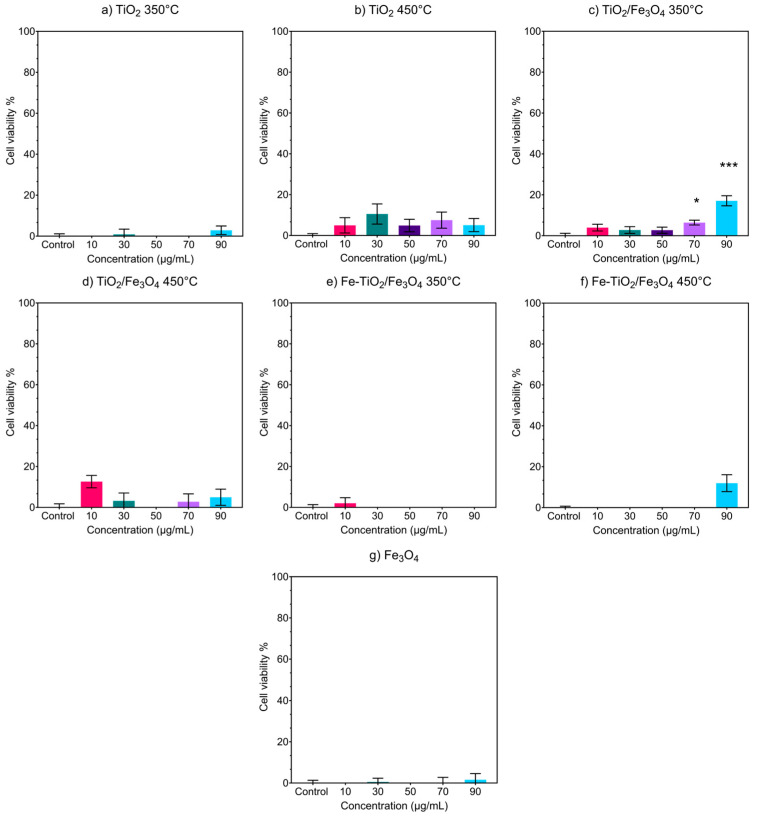
LDH cell viability assays of (**a**) TiO_2_ at 350 °C, (**b**) TiO_2_ at 450 °C, (**c**) TiO_2_/Fe_3_O_4_ at 350 °C, (**d**) TiO_2_/Fe_3_O_4_ at 450 °C, (**e**) Fe-TiO_2_/Fe_3_O_4_ at 350 °C, (**f**) Fe-TiO_2_/Fe_3_O_4_ at 450 °C, and (**g**) Fe_3_O_4_. Cell viability by LDH assay was determined after 24 h of exposure to increasing concentrations of NPs. Each bar represents the average value of 3 different experiments ± SEM; * *p* < 0.05, *** *p* < 0.001 vs. untreated cells. ANOVA, Bonferroni post hoc.

LDH cell viability results are shown in Figure 10. TiO_2_ nanoparticles at 350 °C and 450 °C (Figure 10a,b) show no increase in cell death associated with LDH release by cell membrane damage. The TiO_2_/Fe_3_O_4_ 350 °C composite (Figure 10c) exhibits an increase at 70 and 90 µg/mL of 6.4–17.08% cell death. The composite at 450 °C (Figure 10d) shows no effect. Similarly, the iron-doped composites at 350 °C and 450 °C (Figure 10e,f) show no difference in treatments compared to the control. The magnetite sample (Figure 10g) also does not cause an augmentation of cell death.

### 3.9. Holotomography

**Figure 11:**
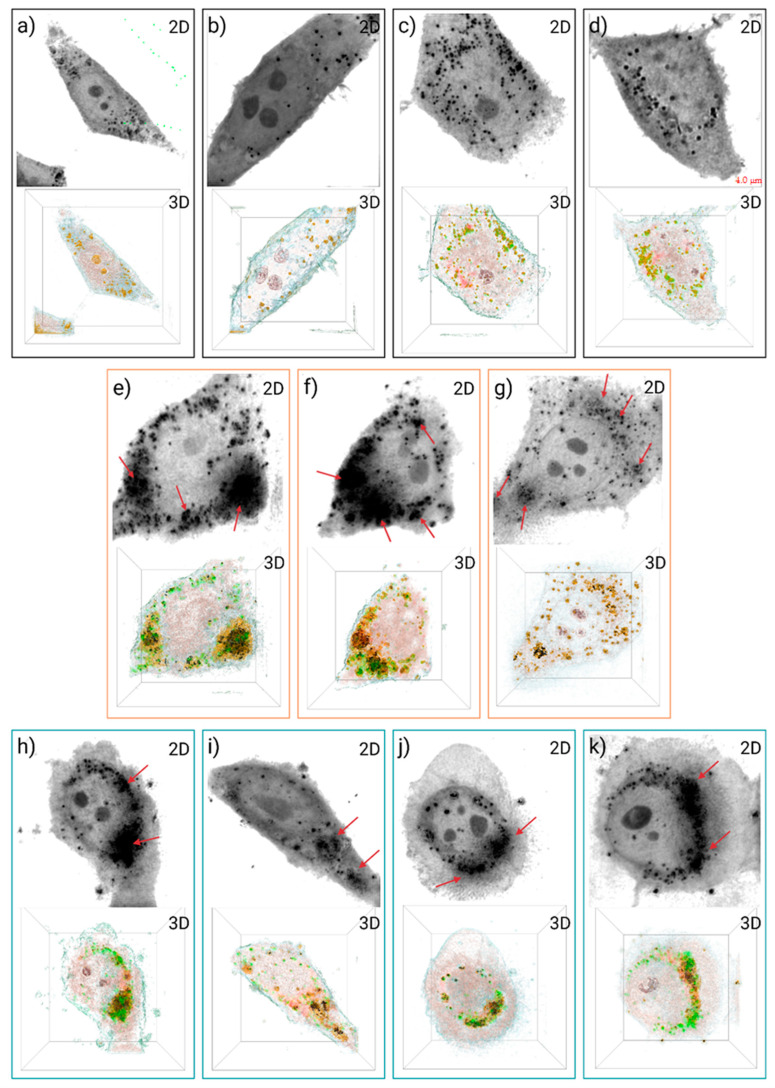
Three-dimensional and two-dimensional holotomographic images of control cells (**a**–**d**), TiO_2_ at 450 °C (**e**–**g**), and TiO_2_/Fe_3_O_4_ at 450 °C (**h**–**k**); red arrows indicate NMs agglomerates. All images were taken at 4 μm zoom.

Figure 11a–d show holotomography images of control cells. The images consist of 2D holotomograms showing a gray RI gradient, with the darkest areas indicating a higher RI, and a 3D image composed of multiple regions of RI colored using the Tomocube software (version HT-X1). The 2D image distinguishes various structures based on their RI, such as the cell membrane, cell nucleolus, and black dots indicating lipid droplets. Three-dimensional holotomography was employed to observe structures such as yellow-colored lipid droplets. The presence of lipid droplets was verified using a green fluorophore superimposed in the yellow areas, as shown in Figure 11c,d. The presence of the fluorophore on the yellow-colored structures confirms that the spherical structures distributed inside the cell observed by holotomography correspond to lipid droplets.

Figure 11e–g illustrate the exposure of TiO_2_ NMs at 450 °C. NMs concentration was 10 µg/mL due to light scattering effects caused by agglomerates of NMs at higher concentrations. Moreover, 2D holotomography shows amorphous structures with high RI at the edges of the cell, along with smaller circular structures corresponding to lipid droplets. NM structures are marked with red arrows for visual aid. Additionally, 3D holotomography reveals the presence of irregular black structures that correspond to NMs interacting with the cellular structure. Both holotomography staining and fluorescence disclose the presence of lipid droplets in areas where NMs are present. The 3D image also displays the interaction of lipid droplets and NMs, where an accumulation of lipid droplets is generated around the NMs agglomerates.

The TiO_2_/Fe_3_O_4_ composites calcined at 450 °C are shown in Figure 11h–k. The exposure to the composite resulted in cells losing their geometric morphology. Moreover, 2D holotomography shows irregular zones with a higher RI, represented in black in the 3D image, corresponding to NMs interacting with the cell. Most of the fluorophore and yellow zones attributed to the lipid droplet in the areas where NMs occur were corroborated. The high internalization of NMs within cells can be observed by holotomographic microscopy. The observed alterations in cell morphology and the internalization of NMs indicate damage and possibly cell death. Holotomography analysis can identify adverse effects on cells that traditional cell viability assays may miss.

## 4. Discussion

Hemolysis tests verify that TiO_2_, Fe_3_O_4_, and the composite NMs are highly biocompatible with the red blood cells when calcinated. Maintaining a level of hemolysis below 2% corresponds to a non-hemolytic material; this is based on the American Society for Testing and Materials (ASTM) F756-13 [50,51]. The change in exposure conditions, such as the use of light or the elimination of the blood plasma content, did not mean a relevant increase in the hemolytic capacity of the NMs. However, the possible residual content of synthesis residues like CTAB could be the predominant factor in defining the hemolytic aspect of non-thermally treated NMs. CTAB’s impact on red blood cell cytotoxicity has been previously observed in gold-based NMS. Gold NMs with a CTAB coating caused higher hemolysis than uncoated and citrate-coated materials [52]. Hemolytic properties of gold NMs coated with poly(sodium-p-styrene sulfonate) and polyethylene glycol were also lower compared to CTAB coatings [53,54]. CTAB residue release through mesoporous silica materials was considered one of the possible mechanisms by which hemolytic activity was increased [55]. Cell membrane disruption and pore formation in erythrocytes due to electrostatic interactions with CTAB is considered one of the mechanisms by which NMs covered by this surfactant can generate hemolysis [56].

Furthermore, Z potential analysis has revealed that under salt-rich media, NMs possess low stability, indicating that the NMs could suffer from aggregation and precipitation, reducing their contact with erythrocytes, which helps to reduce their hemolytic activity as observed in a study where Ag nanoparticles with low agglomeration caused higher hemolysis as compared to the increased accumulation [57].

It should be noted that some studies have determined that NMs can interfere with colorimetric assays such as MTS. These phenomena have been observed with dextran-coated maghemite and magnetite using MTS, where between 1 × 10^−3^ and 10 μg/mL, no effect on absorbance was detected on a cell-free assay. However, at 100 μg mL^−1^, both NMs increased background absorbance even on assays free of MTS [58]. On cell-free MTT assays, TiO_2_ Degussa-P25 has been shown to produce photocatalytic interactions with the colorimetric reagent, reducing the MTT into formazan proportional to the TiO_2_ concentration, revealing increases of cell viability up to 14%, causing underestimation of toxicity [59]. To avoid false determinations of cell viability, it is suggested that the NMs should be previously tested in cell-free environments to identify possible interferences [59].

Cell viability results showed that the materials that affected cell viability reduction were TiO_2_ at 350 °C, TiO_2_ at 450 °C, TiO_2_/Fe_3_O_4_ at 350 °C, and Fe_3_O_4_. However, these last two materials did not show a decrease in viability in a concentration-related manner. On the other hand, the rest of the NMs showed no difference between the treatments and control. An LDH cell viability test demonstrated substantial variability where all materials did not cause cell death except for TiO_2_/Fe_3_O_4_ 350 °C, which was increased at 70 and 90 µg mL^−1^. NMs can interfere in tests by absorbing wavelengths in the spectrum used to measure the MTT absorption, prevent reactions, absorb test reagents, and release ions to alter the catalytic activity of the cells, as well as interfere with other tests such as LDH by absorbing or inactivating LDH protein [60]. NM interference has also been identified in different cell assays, such as alamarBlue and neutral red [61]. Some authors return to the centrifugation of good contents after exposure to eliminate the presence of NMs and reduce interference [62].

In contrast to cell viability results, holotomographic microscopy was employed, which helped to differentiate the different cellular structures based on their refractive index [22,23,24]. This allowed us to determine structures, such as lipid droplets and NMs, which formed amorphous structures and modified HepG2 morphology, possibly due to the incorporation of these NMs into cell interiors. Multiple routes, such as endocytosis mediated by clathrin or caveolin, macropinocytosis, pinocytosis, phagocytosis, and independent routes of clathrin and caveolin, can internalize NMs into cells [63,64,65]. These routes will depend on the physicochemical properties of NMs, such as size, shape, topography, superficial load, hydrophobicity functional groups, and hydrophilicity [63,64,65]. Structures whose dimensions are less than 200 nm and have a positive surface are favored by endocytosis mediated by clathrin. NMs smaller than 50 nm between +15 to −15 mV are transported easily by endocytosis mediated by the caveolin. On the other hand, NMs greater than 250 nm are transported through macropinocytosis [65].

Further investigations using holotomographic microscopes have observed the internalization of different nanostructures and how these are distributed in cells [66,67]. For example, the internalization of gold nanoparticles resulted in lipid droplet generation within macrophages, indicative of a morphology activation and the conversion of macrophages to phagocytes [24]. On the other hand, the exposure of macrophages to quantum graphene dots increased the number of lipid droplets, cellular volume, and dry mass due to the incorporation of NMs [68].

NMs can induce toxic effects at the cellular level through electrostatic interactions, such as association with the cell membrane. The dissolution of toxic ions that can join proteins and enzymes inhibits cellular functions or the generation of ROS, causing peroxidation lipidic, DNA oxidation, oxidative stress, inflammation, and damage to proteins [69,70]. Liver cells exposed to NMs may suffer from inflammation, oxidative stress, and cellular death. Cancer cell lines have also been observed to have greater sensitivity to NMs than standard cell lines [12].

## 5. Conclusions

Our results demonstrated how the physicochemical characteristics influence the toxicity of NMs. In this study, the most prominent property was related to NMs surface characteristics, where it was possible to relate that residual elements of synthesis such as surfactants increase human erythrocytes cell lysis, while thermally treated NMs result in no hemolytic effect that surpasses the 2% of hemolysis. Modifying nanoparticle characteristics, such as the composition, presence, or absence of dopants (Fe); hydrodynamic size; surface charge (Z potential); and their thermal treatment, did not appear to influence their biocompatibility with red blood cells to such an extent, keeping them non-hemolytic under the 2% threshold.

Cell viability studies and holotomographic microscopy allowed us to contrast two tests, where through cell viability (MTS and LDH), it was observed that NMs did not cause a broad reduction in cell viability and where characteristics such as composition, doping, and thermal treatment did not show a substantial difference of biocompatibility levels. On the other hand, holotomography showed a high incorporation of NMs in the cell structure, altering its morphological properties, which can lead to cell death; with this assay, no difference between NM characteristics was found related to NM incorporation in cells.

The results give us a greater perspective on the safety of using composites formed by TiO_2_ and Fe_3_O_4_ for applications such as water remediation, emphasizing the properties they can acquire from their synthesis method and how this influences their toxic activity. Alternatives such as synthesis processes that avoid using harmful substances for humans can help reduce the potential toxicity of the synthesized NMs. On the other hand, in vitro tests suppose effective alternatives to evaluate the toxicity of NMs; however, studies with more complex cellular environments, which include, for example, other cells present in the liver, would allow us to obtain a better perception of the interaction of these NMs and how its toxicity mechanisms can affect cells present in an organ to differing degrees.

Holotomographic microscopy offered novel alternatives to observe the interaction of NMs with HepG2 cells, showing their high inclusion in the cell structure. Holotomography can be exploited to visualize the transfer and interaction of NMs in the cell environment. In turn, their results could be complemented with other equipment, such as atomic force microscopy, providing parameters referring to the mechanical properties of cells and how the presence of NMs can modify them, also supporting or contrasting the results obtained from classical cell viability test that their interaction with nanomaterials can alter.

## Figures and Tables

**Table 1:** Comparison of crystallite size between Scherrer and Williamson–Hall equations.

NMs	Scherrer (nm)	Williamson–Hall (nm)
TiO_2_ 350 °C	8.0	6.1
TiO_2_ 450 °C	13.1	11.2
TiO_2_/Fe_3_O_4_ 350 °C	8.6	8.4
TiO_2_/F_3_O_4_ 450 °C	10.9	9.6
Fe-TiO_2_/Fe_3_O_4_ 350 °C	8.3	8.7
Fe-TiO_2_/Fe_3_O_4_ 450 °C	13.4	11.9

**Table 2:** Characterization of the NMs in the different suspended media.

NMs	Water		Saline Solution (0.9%)		DMEM Medium			
					0 h		24 h	
	Hydrodynamic Diameter (nm)/PDI	Zeta Potential (mV)	Hydrodynamic Diameter (nm)/PDI	Zeta Potential (mV)	Hydrodynamic Diameter (nm)/PDI	Zeta Potential (mV)	Hydrodynamic Diameter (nm)/PDI	Zeta Potential (mV)
Fe_3_O_4_	1125 ± 48.22/ 0.227	−17.9 ± 4.87	888.53 ± 176.37/ 0.107	−13.84 ± 7.48	1042.47 ± 94.53/ 0.683	−172.67± 30.66	512.63 ± 7.85/ 0.663	−244.66 ± 37.80
TiO_2_ 350 °C	43.14 ± 1.02/ 0.275	−2.19 ± 1.19	343.53 ± 77.26/ 0.192	−4.62 ± 6.34	439.83 ± 3.20/ 0.175	−11.73 ± 1.30	433.27 ± 16.14/ 0.398	−10.43 ± 0.21
TiO_2_ 450 °C	49.87 ± 0.69/ 0.244	−2.30 ± 0.61	247.87 ± 33.16/ 0.224	−4.98 ± 7.80	765.3 ± 57.23/ 0.364	−	337.73 ± 7.45/ 0.152	−13.4 ± 0.91
TiO_2_/Fe_3_O_4_ 350 °C	118.9 ± 13.07/ 0.318	−4.34 ± 0.218	293.56 ± 72.29/ 0.187	−0.203 ± 4.25	534.43 ± 22.70/ 0.148	−152.33 ± 21.93	367.73 ± 4.94/ 0.177	−196.33 ± 20.81
Fe-TiO_2_/Fe_3_O_4_ 350 °C	145.43 ± 37.94/ 0.237	−6.41 ± 2.39	201.17 ± 54.25/ 0.204	−0.27 ± 4.85	929.73 ± 22.30/ 0.230	−165.67 ± 34.12	436.53 ± 35.47/ 0.52	−245.67 ± 28.36

Samples were analyzed once per triplicate. Results are expressed as mean ± SD.

## Data Availability

No new data were created or analyzed in this study. Data sharing is not applicable to this article.

## Supplementary Materials

The following supporting information can be downloaded at: https://www.mdpi.com/article/10.3390/nano13212824/s1, Table S1: Determination of biocompatibility through hemolysis assay; Figure S1: EDX elemental spectra of TiO_2_; Figure S2: EDX elemental spectra of Fe_3_O_4_; Figure S3: EDX elemental spectra of TiO_2_/Fe_3_O_4_ composite; Figure S4: EDX elemental mapping zone and elemental mapping of (a,b) TiO_2_, (c,d) Fe_3_O_4_, and (e,f) TiO_2_/Fe_3_O_4_; Figure S5: Photocatalytic degradation of paracetamol under UV light; Figure S6: Hemolysis assay of NMs interaction with (a) blood and plasma in a dark environment, (b) blood and plasma under light exposure, (c) blood without plasma under a dark environment, and (d) blood without plasma under light exposure.
