# Supplementary material for: Biocompatibility Evaluation of TiO2, Fe3O4, and TiO2/Fe3O4 Nanomaterials: Insights into Potential Toxic Effects in Erythrocytes and HepG2 Cells
Source: Nanomaterials (Basel). 2023 Oct 25;13(21):2824. doi: 10.3390/nano13212824 (PMC10648038; doi:10.3390/nano13212824)
Supplement: Supplementary file 1 [file nanomaterials-13-02824-s001.zip › nanomaterials-2663225-supplementary.pdf]

Biocompatibility evaluation of TiO_2, Fe_3O_4, and TiO_2/Fe_3O_4 nanomaterials: insights into potential toxic effects in erythrocytes and HepG2 cells

Luis Paramo[1], A. Jiménez-Chávez[2], Iliana E Medina-Ramirez[3], Harald N. Böhnel[4], Luis Escobar-Alarcón[5], Karen Esquivel[1]

[1] *División de Investigación y Posgrado, Facultad de Ingeniería, Universidad Autónoma de Querétaro, Cerro de las Campanas, Santiago de Querétaro 76010, Mexico.*

[2] *Departamento de Toxicología, Centro de Investigación y de Estudios Avanzados del IPN (CINVESTAV-IPN), Ciudad de México, México*

[3] *Department of Chemistry, Universidad Autónoma de Aguascalientes, Aguascalientes 20131, Mexico.*

[4] *Centro de Geociencias, Universidad Nacional Autónoma de México, Campus Juriquilla. Blvd. Juriquilla, 3001, Santiago de Querétaro 76230, Mexico.*

[5] *Departamento de Física, Instituto Nacional de Investigaciones Nucleares, Carr. México-Toluca, La Marquesa, Ocoyoacac 52750, Mexico.*

Electronic Supplementary Material

Table S1. Determination of biocompatibility through hemolysis assay.

Tube	Concentration (μg/mL)	NMs (μL)	PSS (mL)	distilled water (mL)	Blood (μL)
Control +	-	-	0	10	100
Control -	-	-	10	0	100
1	10	100	9.9	0	100
2	30	300	9.7	0	100
3	50	500	9.5	0	100
4	70	700	9.3	0	100

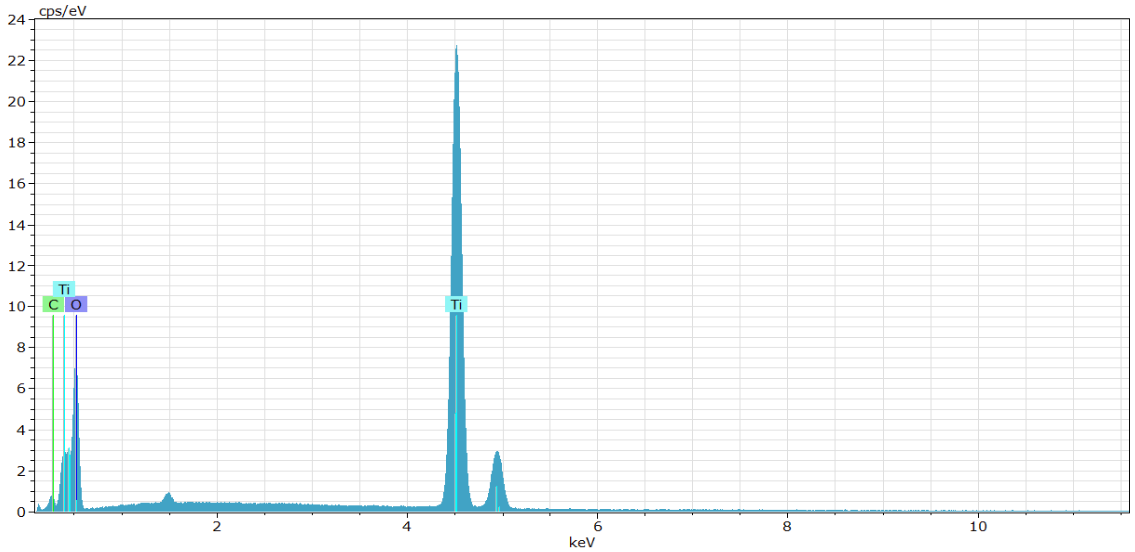

Figure S1. EDX elemental spectra of TiO2

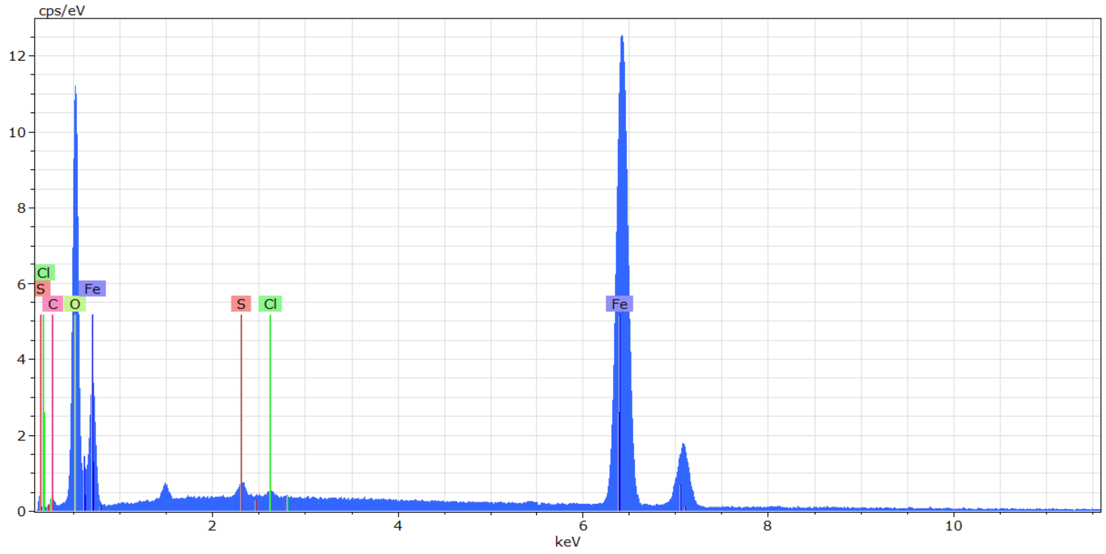

Figure S2. EDX elemental spectra of Fe3O4

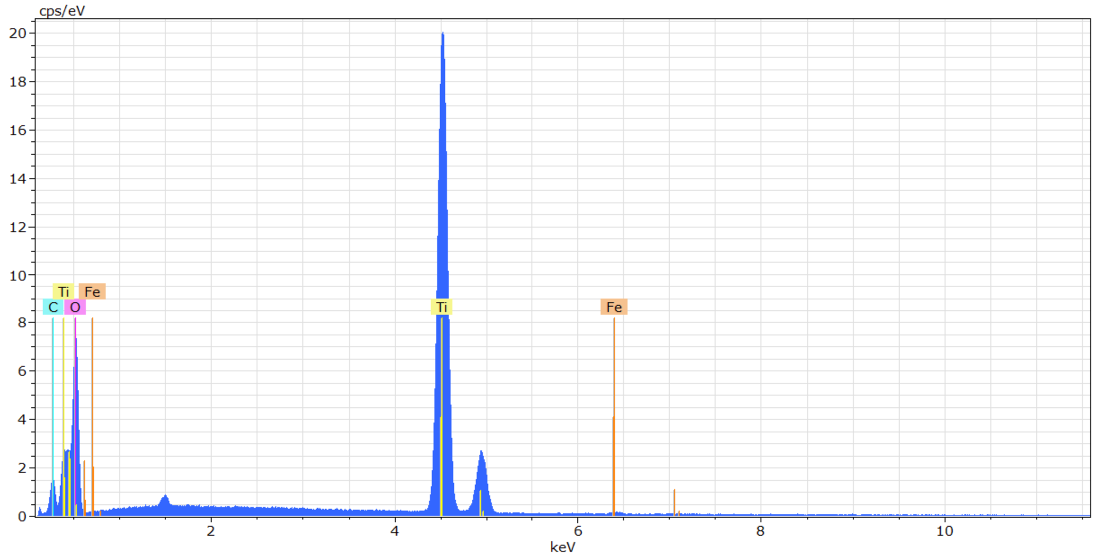

Figure S3. EDX elemental spectra of TiO2/Fe3O4 composite.

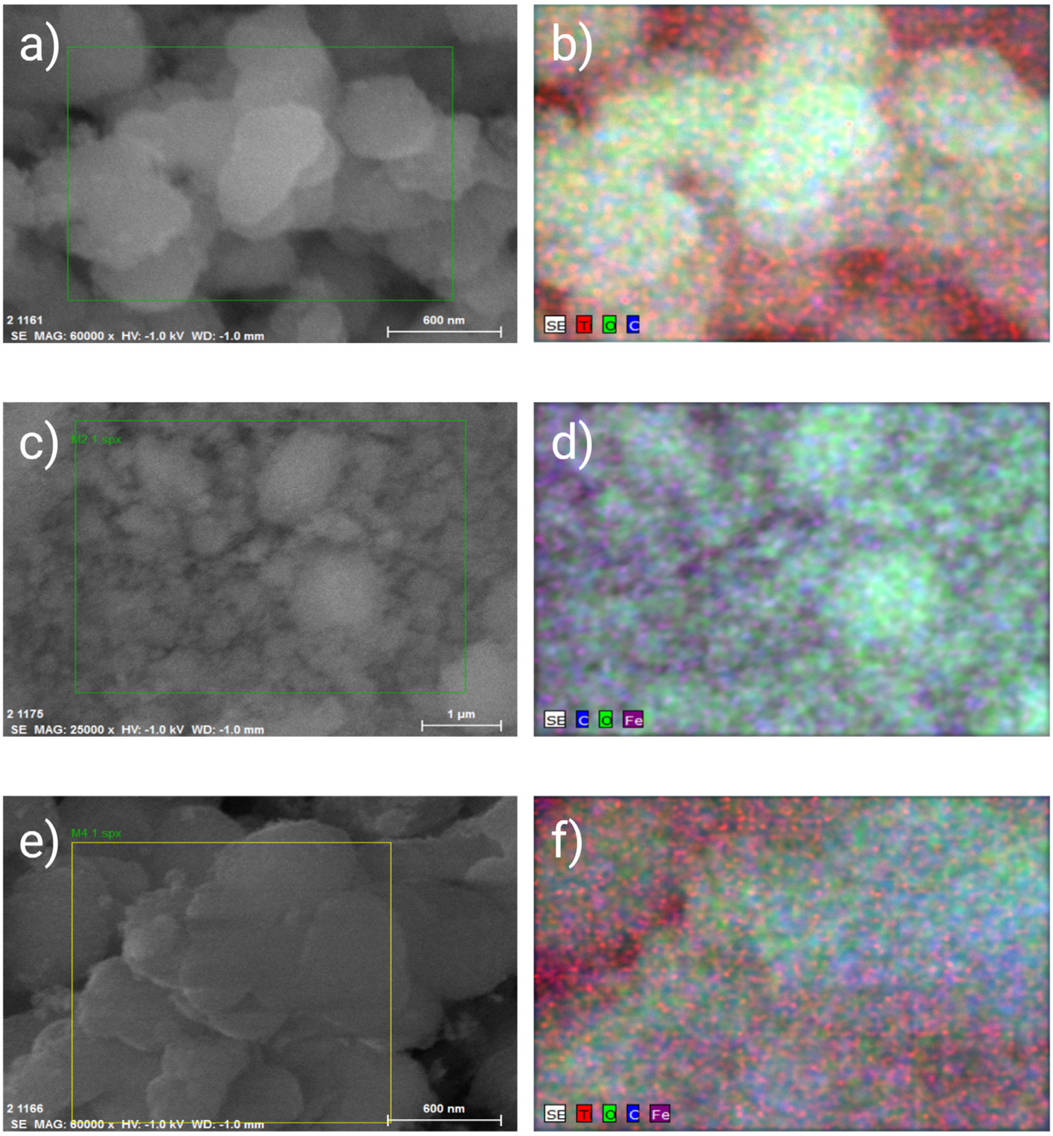

Figure S4. EDX elemental mapping zone and elemental mapping of a,b) TiO2, b,c) Fe3O4 and e,f) TiO2/Fe3O4.

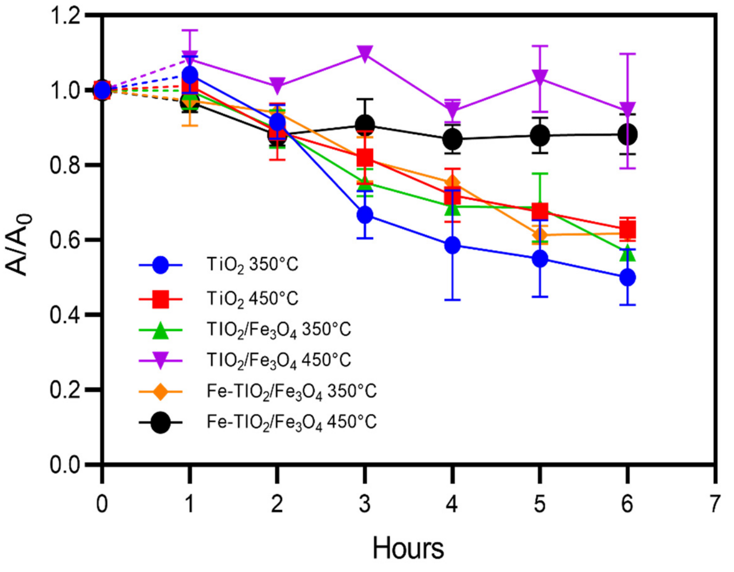

Figure S5. Photocatalytic degradation of paracetamol under UV light.

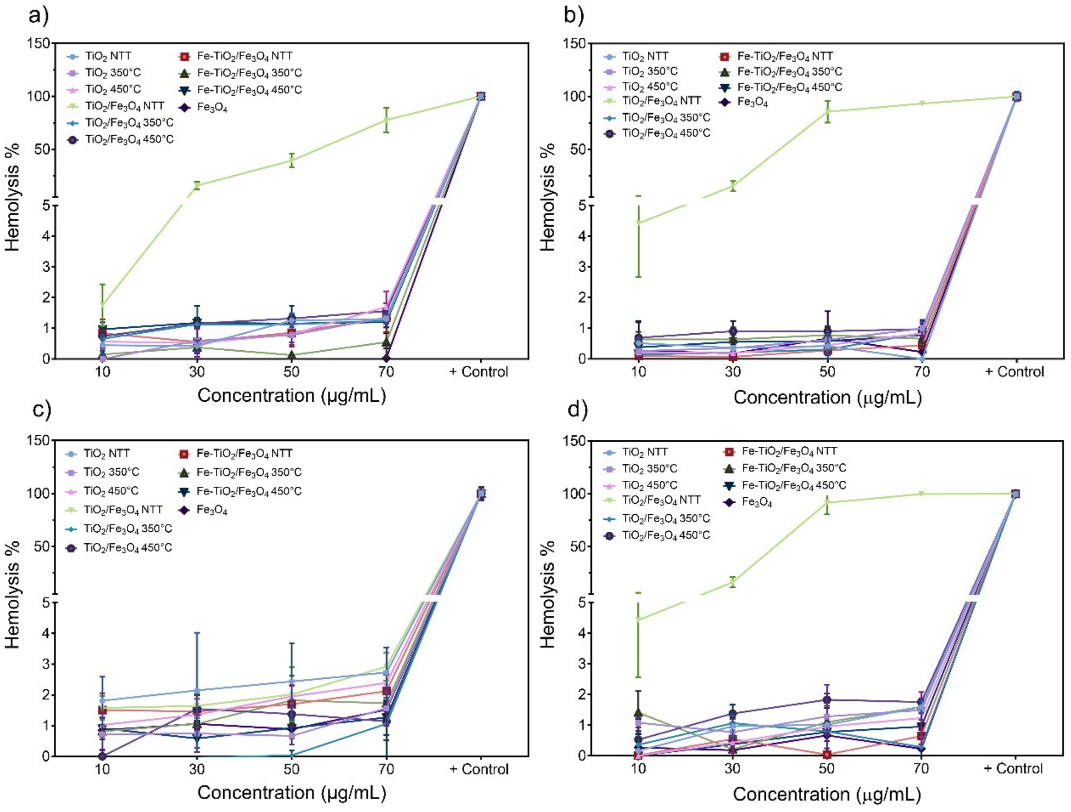

Figure S6. Hemolysis assay of NMs interaction with a) blood and plasma in a dark environment, b) blood and plasma under light exposure, c) blood without plasma under a dark environment, and d) blood without plasma under light exposure.
